# Three new species of the leafhopper genus *Tautoneura* Anufriev (Hemiptera, Cicadellidae, Typhlocybinae) from China

**DOI:** 10.3897/zookeys.83.1178

**Published:** 2011-02-25

**Authors:** Yuehua Song, Zizhong Li, Jing Xiong

**Affiliations:** Institute of South China Karst, Guizhou Normal University, Guiyang, Guizhou 550001, China; Institute of Entomology, Guizhou University, Guiyang, Guizhou 550025, Chin

**Keywords:** Homoptera, *Tautoneura*, new species, morphology, taxonomy, China

## Abstract

In the present paper, three new species are added to the genus Tautoneura Anufriev from China, Tautoneura baiyunshana **sp. n.**, Tautoneura caoi **sp. n.** and Tautoneura yunnanensis **sp. n.** A key to species recorded from China is provided.

## Introduction

The leafhopper genus Tautoneura Anufriev (1969) belongs to the tribe Erythroneurini (Typhlocybinae) with Tautoneura tricolor Anufriev, 1969 as its type species. The genus consists of fifty-one species distributed in the Oriental and Palaearctic Regions. So far, twelve species in Tautoneura have been reported from China. A key to Chinese species and a species checklist of Tautoneura from China are provided. In this paper, three new species are described and illustrated from Henan, Yunnan and Gangsu Provinces of China. All specimens examined are deposited to the Institute of South China Karst, Guizhou Normal University (ISCK) and Insititute of Entomology, Guizhou University (IEGU), Guiyang, China.

## Materials and methods

The specimens were obtained by sweep net method and were studied under Olympus SZX7 and CX41 microscopes. Morphological techniques and terminology follow 4and Dmitriev (2006). Measurements of the new species are given in millimeters; body length is measured from the apex of head to apex of forewing in repose.

## Taxonomy

### Tautoneura

Anufriev, 1969

Tautoneura Anufriev 1969: 186–188
Erythroneura (Balia) Dworakowska, 1970 (Dworakowska 1977: 290)
Erythroneura (Havelia) Ahmed, 1971 (Dworakowska 1977: 290; Dworakowska 1980: 182)

#### Type species:

Tautoneura tricolor Anufriev, 1969

#### Description.

Body small, about 2.0–3.0 mm, usually yellow or light yellow. Head bluntly produced medially, slightly narrower than pronotum or equal to greatest width of pronotum. Median length of vertex equal to or longer than length between eyes. Some species with more rounded anterior margin of vertex. Pronotum broad, often with irregular patches or spots; scutellum nearly triangular. Forewings usually with red markings or spots.

Pygofer lobe broad, usually with several macrosetae at basal lower angle and some short stout setae in distal part of lobe on inner surface, peg-like. Dorsal appendage of pygofer long, tapering apically, movably articulated with pygofer lobe. Some species have ventral appendage. Aedeagus usually with large dorsal apodeme and one or two pairs of processes of variable length at apex of shaft. Style slender, with slim “neck” subapically, and prominent preapical lobe. Connective nearly M- or Y-shaped, central lobe well developed, as long as or little shorter than lateral arms. Shape of anal tube appendage diverse, but that of most species hook-like at apex.

#### Distribution.

Palaearctic and Oriental Regions.

#### Key to males of Tautoneura from China.

**Table:** 

1	Vertex with two longitudinal red stripes that converge at anterior margin midpoint of vertex	Tautoneura mori
–	Vertex without convergent longitudinal stripes	2
2	Aedeagal shaft without processes	3
–	Aedeagal shaft with one or more processes	4
3	Aedeagus preatrium with one pair of processes	Tautoneura arachisi
–	Aedeagus preatrium without processes (Figs 16, 17)	Tautoneura yunnanensis sp.n.
4	Aedeagal shaft with two pairs of processes	5
–	Aedeagal shaft with only one or one pair of processes	7
5	Aedeagal shaft process small, short, with one or more teeth	6
–	Aedeagal shaft process long, slim, finger-like	Tautoneura formosa
6	Abdominal apodemes very slim, not extended beyond posterior margin of 3rd sternite (Fig. 3)	Tautoneura baiyunshana sp. n.
–	Abdominal apodemes expanded distinctly, extended to 5th sternite	Tautoneura longiprocessa
7	Aedeagal shaft with single, irregular process	Tautoneura sinica
–	Aedeagal shaft with pair of processes	8
8	Processes arising from apex or sub-apex of aedeagal shaft	9
–	Processes arising from base or sub-base of aedeagal shaft	13
9	Processes arising from apex of aedeagal shaft	10
–	Processes arising from sub-apex of aedeagal shaft	Tautoneura prima
10	Apex of style long and slim, slightly curved	Tautoneura fusca
–	Apex of style truncate or short and broad	11
11	Pronotum with nearly rectangular red spot medially	Tautoneura choui
–	Pronotum without red medial spot	12
12	Forewing with three round red spots	Tautoneura tripunctula
–	Forewing with many orange-yellow markings, some areas with red spots or stripes	Tautoneura multimaculata
13	Aedeagus dorsal appendage bifurcate at apex (Fig. 22)	Tautoneura caoi sp. n.
–	Aedeagus dorsal appendage not bifurcate at apex	Tautoneura albida

(Note: the key does not include Tautoneura takaonella Mats. 1932, as only females of the species are known.)

#### Tautoneura baiyunshana

Song, Li & Xiong sp. n.

urn:lsid:zoobank.org:act:87B96B3F-18A7-4F49-A9B4-B3D42863ECC4

Figures 1–9 

##### Description.

**Figures 1–9.**
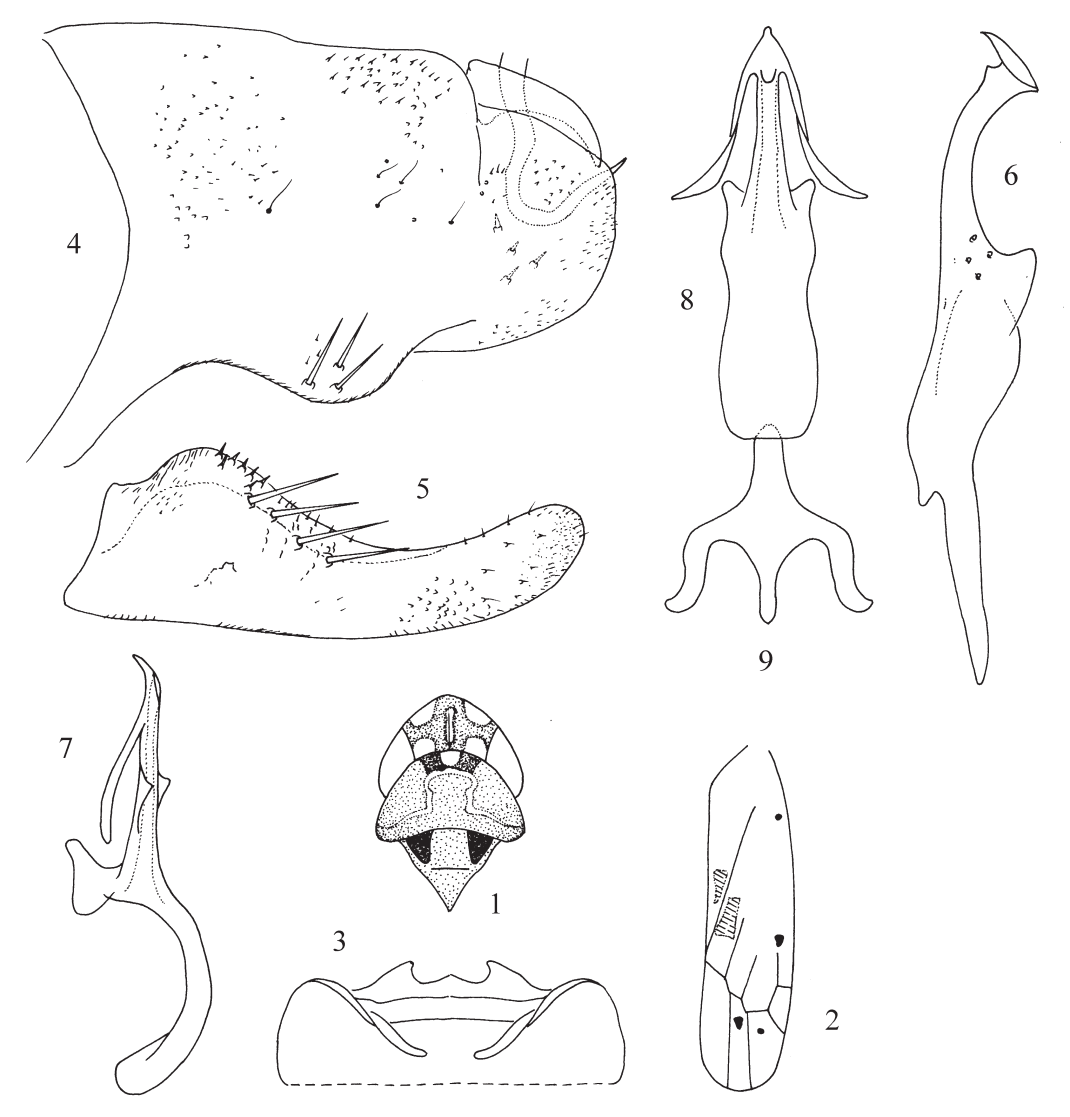
Tautoneura baiyunshana Song, Li & Xiong, sp. n. **1** Head and thorax, dorsal view **2** Forewing **3** Abdominal apodemes **4** Pygofer lobe, lateral view **5** Subgenital plate **6** Style **7** Aedeagus, lateral view **8** Aedeagus, ventral view **9** Connective.

Body brownish yellow or brown testaceous. Head (Fig. 1) narrower than pronotum. Crown (Fig. 1) anteriorly produced medially, coronal suture distinct. Vertex (Fig. 1) median length little shorter than width between two eyes. Vertex and pronotum (Fig. 1) with milky yellow stripes. Eyes (Fig. 1) brownish yellow. Scutellum (Fig. 1) brownish yellow, basal triangles darker. Forewing (Fig. 2) brownish yellow, semitransparent, with four irregular dark spots and with two broad orange red patches near claval suture.

Abdominal apodemes (Fig. 3) small, not exceeding hind margin of 3rd sternite.

Pygofer (Fig. 4) broad, with three macrosetae at baso-lateral angle and a few sparse long fine setae. Pygofer microtrichia conspicuous, well developed. Pygofer dorsal appendage simple, not extended beyond pygofer apex, curved ventrally. Anal tube appendage (Fig. 4) hook-like apically. Subgenital plate (Fig. 5) lateral margin distinctly widened subbasally, with four macrosetae near median and with several short rigid setae along upper margin of sub-basal part. Style (Fig. 6) apex expanded, preapical lobe prominent. Connective (Fig. 9) nearly Y-shaped, central lobe and lateral arms slender; stem well developed, compressed. Aedeagal shaft (Fig. 7) almost straight, with pair of long processes arising near apex, another pair of short processes at middle area of shaft, triangular, lamellate in lateral view. Gonopore (Figs 7, 8) subapical and on ventral margin. Dorsal apodeme (Fig. 7) short; preatrium (Figs 7, 8) long and slim, about as long as or little longer than shaft.

##### Measurement.

Body length males 2.5~2.6 mm, females 2.6~2.8 mm.

##### Host Plant.

Unknown.

##### Type material.

*Holotype*, male, China: Henan Province, Mt. Baiyunshan, 1300~1400 m, 17 August 2008, coll. Can Li. *Paratypes*: two males, ten females, same data as holotype.

##### Remarks.

The new species is similar to Tautoneura longiprocessa Song & Li (2008), but can be distinguished from the latter by the paired long processes arising from subapex of shaft (Figs 7, 8); gonopore (Figs 7, 8) subapical; preatrium (Fig. 7) slim, longer than aedeagal shaft.

##### Etymology.

The new species is named after its type locality: “Baiyunshan”, Henan Province.

#### Tautoneura yunnanensis

Song, Li & Xiong sp. n.

urn:lsid:zoobank.org:act:A8E8704B-EE04-457B-BA5E-1E00413FCDC7

Figures 10–18 

##### Description.

**Figures 10–18.**
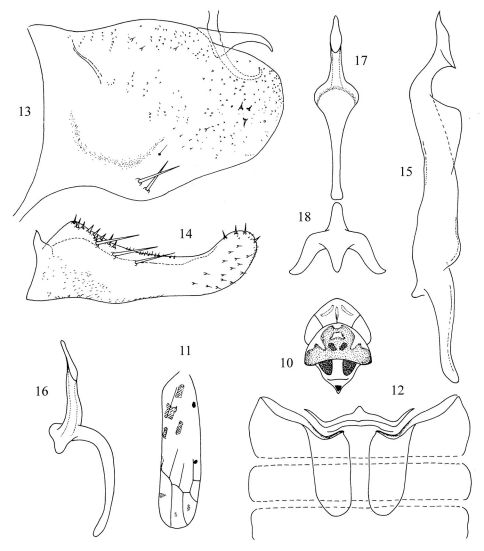
Tautoneura yunnanensis Song, Li & Xiong, sp. n. **10** Head and thorax, dorsal view **11** Forewing **12** Abdominal apodemes **13** Pygofer lobe, lateral view **14** Subgenital plate **15** Style **16** Aedeagus, lateral view **17** Aedeagus, ventral view **18** Connective.

Body brownish yellow. Crown (Fig. 10) fore margin strongly produced and angulate medially. Coronal suture (Fig. 10) long, milky, nearly extended to 4/5 middle length of vertex. Two slim curving milky stripes situated at sides of coronal suture symmetrically. Eyes (Fig. 10) grey testaceous. Pronotum (Fig. 10) with two small orange red spots medially and broad area around them brownish. Scutellum (Fig. 10) light brown, with orange yellow spot at apex; basal triangles orange yellow. Forewing (Fig. 11) with four orange markings around claval suture and several brownish or blackish brown spots.

Abdominal apodemes (Fig. 12) large, broad, reaching 5th sternite.

Pygofer lobe (Fig. 13) broad, with two macrosetae at basal lower angle and numerous fine setae or microsetae distributed on lateral surface. Pygofer microtrichia distinct. Pygofer dorsal appendage expanded at base and tapering towards apex, bent ventrally. Anal tube appendage (Fig. 13) slim, hook-like apically. Subgenital plate (Fig. 14) with three basal macrosetae, expanded subbasally and with several peg-like short setae. Style (Fig. 15) quite long, apex expanded obviously; preapical lobe large, prominent. Connective (Fig. 18) Y-shaped, lateral arms strong, central lobe and stem well developed. Aedeagal shaft (Figs 16, 17) almost straight and short in lateral view, its base conspicuously expanded in ventral view; without any process. Gonopore (Figs 16, 17) broad, apically. Aedeagus dorsal apodeme (Fig. 16) short and small; preatrium (Figs 16, 17) much longer than aedeagal shaft.

##### Measurement.

Body length males 2.2~2.3 mm.

##### Host plant.

Unknown.

##### Type material.

*Holotype*, male, China: Yunnan Province, Lijiang, 16 July 2010, coll. CAN LI. *Paratypes*: one male, same data as holotype; one male, China: Yunnan Province, Mengla County, 18 July 2008, coll. YUEHUA SONG.

##### Remarks.

The new species is similar to Tautoneura misrai Dworakowska (1977), but can be distinguished from the latter by the large and broad, apical gonopore; the aedeagus dorsal apodeme short, not expanded distinctly and the ventral appendage absent; the forewing with four orange patches near claval suture.

##### Etymology.

The new species is named after its type locality: “Yunnan”, China.

#### Tautoneura caoi

Song, Li & Xiong sp. n.

urn:lsid:zoobank.org:act:1CAE0426-23ED-4C0B-AC2A-7013DBAC940F

Figures 19–27 

##### Description.

**Figures 19–27.**
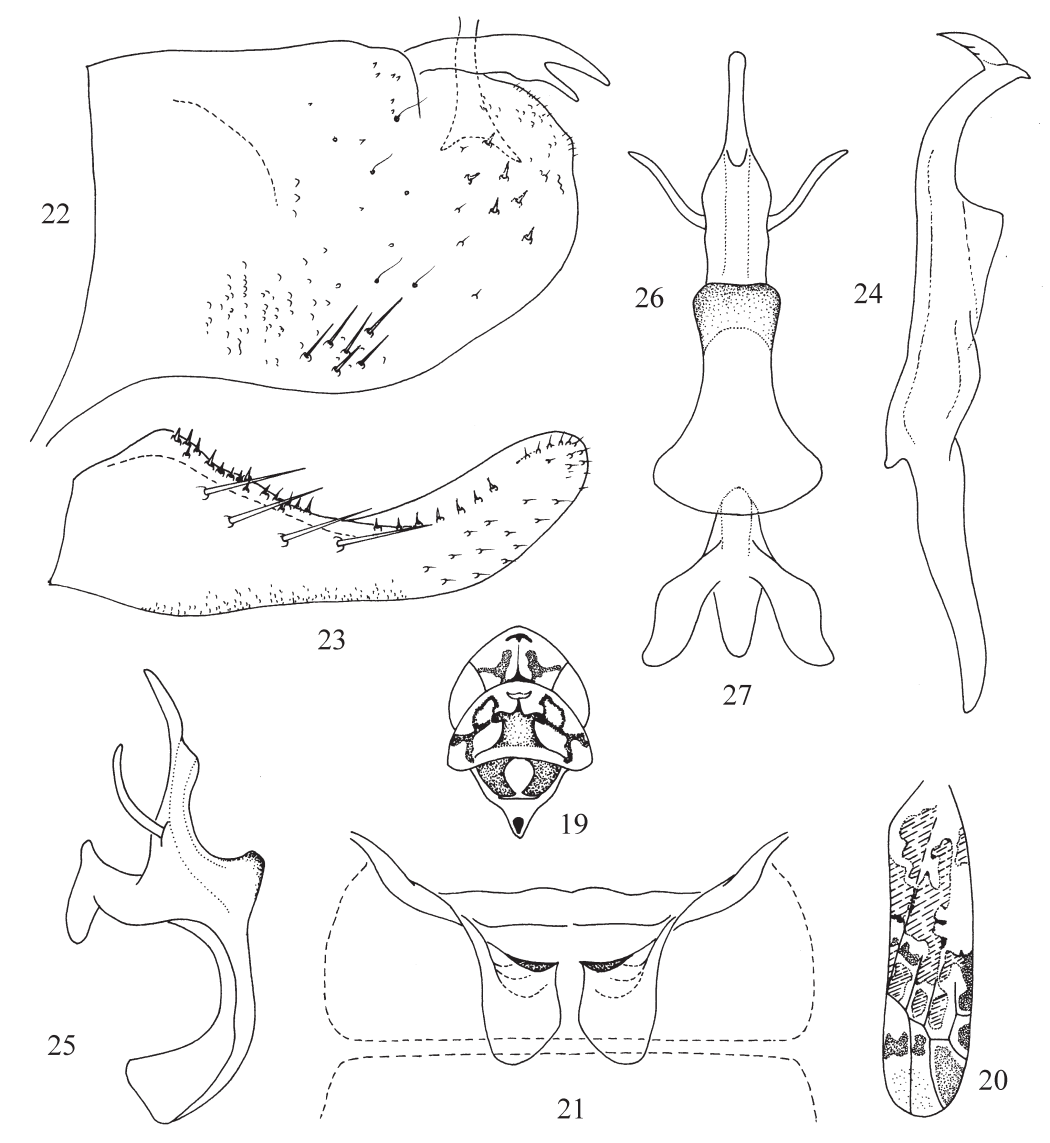
Tautoneura caoi Song, Li & Xiong, sp. n. **19** Head and thorax, dorsal view **20** Forewing **21** Abdominal apodemes **22** Pygofer lobe, lateral view **23** Subgenital plate **24** Style **25** Aedeagus, lateral view **26** Aedeagus, ventral view **27** Connective.

Body yellowish. Structural characters as in Tautoneura baiyunshana sp. n. and Tautoneura yunnanensis sp. n. Vertex and pronotum (Fig. 19) with irregular orange red markings. Eyes grey. Scutellum (Fig. 19) basal triangles orange yellow and apex with dark spot. Forewing (Fig. 20) brownish yellow, semitransparent, with numerous orange yellow markings, some parts with red spots or streak, apex (apical cells) dark brown.

Abdominal apodemes (Fig. 21) broad, extended beyond posterior margin of 3rd sternite.

Pygofer lobe (Fig. 22) broad, with numerous macrosetae, long fine setae and rigid short setae. Pygofer microtrichia inconspicuous. Dorsal appendage bifurcate far from base, extended beyond pygofer apex. Subgenital plate (Fig. 23) with four basal macrosetae and distinct marginal subbasal rigid setae formed continuous row. Style (Fig. 24) slender, apex expended slightly; preapical lobe prominent. Connective (Fig. 27) Y-shaped, two arms strong, central lobe well developed. Aedeagal shaft (Figs 25, 26) short, with pair of lateral processes at sub-base, part between aedeagal shaft and preatrium expanded. Gonopore nearly median, on ventral margin. Dorsal apodeme little longer than that of other two new species. Preatrium long, much longer than aedeagal shaft.

##### Measurement.

Body length males 2.5~2.6 mm, females 2.7~2.8 mm.

##### Host plant.

Ulmus pumila L. (Elm)

##### Type material.

Holotype, male, China: Gansu Province, Zhenyuan County, 19 May 2010, coll. WEI CAO. *Paratypes*: seven males, ten females, same data as holotype.

##### Remarks.

The new species is distinguishable from Tautoneura ahmedi Dworakowska (1977) by the aedeagus preatrium strongly expended at terminal part; the dorsal appengage bifurcate apically and the gonopore near median area of aedeagal shaft.

##### Etymology.

This sepcies is named after its collector.

## Species checklist of Tautoneura

Tautoneura (Havelia) ahmedi Dworakowska 1977. India

Tautoneura (Havelia) alba (Ahmed, 1971) India

Tautoneura (Tautoneura) albida (Dworakowska 1970). China (Guangdong)

Tautoneura (Tautoneura) arachisi (Matsumura, 1916). China (Taiwan)

Tautoneura (Havelia) bellula Dworakowska 1994. Sikkim

Tautoneura (Tautoneura) bena Dworakowska 1981 India

Tautoneura (Havelia) baiyunshana Song, Li & Xiong, sp. n. China (Henan)

Tautoneura (Havelia) caoi Song, Li & Xiong, sp. n. China (Gansu)

Tautoneura (Havelia) choui Ma 1983. China (Shaanxi)

Tautoneura (Tautoneura) deska (Dworakowska 1970). Samoa

Tautoneura (Tautoneura) dubiosa Dworakowska 1981. Nepal

Tautoneura (Tautoneura) dubiosissima Dworakowska 1981. Nepal

Tautoneura (Tautoneura) dukara Dworakowska 1981. India

Tautoneura (Tautoneura) eda Dworakowska 1981. India

Tautoneura (Tautoneura) erythropunctata (Ramakrishnan & Menon, 1973) India

Tautoneura (Tautoneura) ficaria Dworakowska, 1984. India; Singapore

Tautoneura (Tautoneura) formosa (Dworakowska 1970). China (Jiangsu)

Tautoneura (Tautoneura) fusca (Dworakowska 1970). China (Guangdong)

Tautoneura (Tautoneura) incisa Dworakowska 1980. India

Tautoneura (Tautoneura) indefinita (Dworakowska 1970). Samoa

Tautoneura (Tautoneura) japonica Dworakowskam 1972. Japan

Tautoneura (Tautoneura) kira Dworakowska 1981. India

Tautoneura (Tautoneura) klara Dworakowska 1981. India

Tautoneura (Tautoneura) leucothoe (Kirkaldy, 1907). Fiji; Samoa

Tautoneura (Tautoneura) longiprocessa Song and Li 2008. China (Guizhou)

Tautoneura (Havelia) maculosa Sohi, Mann & Shenhmar, 1987. India

Tautoneura (Havelia) manica Thapa, 1989. Nepal

Tautoneura (Tautoneura) marthae (Linnavuori, 1960). Fiji

Tautoneura (Tautoneura) mayarami Mathew & Ramakrishnan, 1996. India

Tautoneura (Tautoneura) misrai Dworakowska 1977. India

Tautoneura (Tautoneura) mori (Matsumura, 1910). China (Shangdong, Anhui, Jiangsu, Zhejiang, Sichuan, Guizhou)

Tautoneura (Tautoneura) mukla Dworakowska 1981. India

Tautoneura (Havelia) multimaculata Song & Li, 2009. China (Guizhou).

Tautoneura (Tautoneura) mureda Dworakowska 1981. Nepal

Tautoneura (Tautoneura) napa Dworakowska 1981. India

Tautoneura (Tautoneura) ochreleuca Thapa, 1984. Nepal

Tautoneura (Havelia) panthera Dworakowska 1994. Sikkim

Tautoneura (Tautoneura) panti Dworakowska 1977. India

Tautoneura (Havelia) pewna Sohi & Mann, 1992. Nepal

Tautoneura (Tautoneura) prima Dworakowska 1979. China (Guizhou)

Tautoneura (Tautoneura) redama Dworakowska 1981. Nepal

Tautoneura (Tautoneura) sanguinalis (Distant, 1918). India

Tautoneura (Tautoneura) secunda Dworakowska 1979. Vietnam

Tautoneura (Tautoneura) sinica (Dworakowska 1970). China (Guangdong, Jiangsu)

Tautoneura (Tautoneura) smocza Dworakowska 1980. India

Tautoneura (Tautoneura) takaonella (Matsumura 1932). China (Taiwan)

Tautoneura (Havelia) tricolor Anufriev 1969. Russia

Tautoneura (Tautoneura) tripunctula (Melichar, 1903). China (Guizhou, Yunnan)

Tautoneura (Havelia) unicolor Dworakowska 1979. Vietnam

Tautoneura (Havelia) yunnanensis Song, Li & Xiong, sp. n. China (Yunnan)

Tautoneura (Tautoneura) zembata Dworakowska 1979. Japan

Tautoneura (Tautoneura) zizypha Thapa, 1984. Nepal

Tautoneura (Tautoneura) zobra Dworakowska 1979. Vietnam

Tautoneura (Havelia) zygina Dworakowska 1994. Sikkim

## Supplementary Material

XML Treatment for Tautoneura
